# Functional Unit Construction for Heat Storage by Using Biomass-Based Composite

**DOI:** 10.3389/fchem.2022.835455

**Published:** 2022-02-07

**Authors:** Jingtao Su, Mengman Weng, Xiang Lu, Weihao Xu, Sha Lyu, Yidong Liu, Yonggang Min

**Affiliations:** ^1^ Department of Polymeric Materials and Engineering, School of Materials and Energy, Guangdong University of Technology, Guangzhou, China; ^2^ Key Laboratory of Polymer Processing Engineering, Ministry of Education, South China University of Technology, Guangzhou, China; ^3^ Key Laboratory of Material Chemistry for Energy Conversion and Storage of Ministry of Education, School of Chemistry and Chemical Engineering, Huazhong University of Science and Technology, Wuhan, China; ^4^ Department of Materials Science and Engineering, Southern University of Science and Technology, Shenzhen, China

**Keywords:** phase change materials, sugarcane-based biomass, three-dimension, polyethylene glycol, SSPCMs

## Abstract

How to construct a functional unit for heat storage by using biomass materials is significant for the exploration of phase change materials (PCMs). In this work, we try to design and construct a functional unit for heat storage by employing a vacuum impregnation method to prepare sugarcane-based shape stabilized phase change materials (SSPCMs) for improving the thermal conductivity of phase change materials (PCMs) and preventing the liquid state leakage of PCMs. The morphologies of the prepared materials are characterized by Scanning electron microscope (SEM) as containing a unique channel structure which is viewed as the key factor for heat storage. X-ray diffractometry (XRD), Fourier transform infrared spectroscopy (FT-IR), and thermogravimetric analysis (TGA) were used to characterize the prepared materials. The results indicated that no chemical reaction occurred between PEG and sugarcane-based biomass during the preparation process and SSPCMs showed great thermal stability. Their thermal properties are measured by using the differential scanning calorimetry (DSC) characterization and show a high melting enthalpy of 140.04 J/g and 94.84% of the relative enthalpy efficiency, illustrating the excellent shape stabilized phase change behavior. Moreover, the highest thermal conductivity of SSPCMs is up to 0.297 W/(mK), which is 28.02% higher than that of the pristine PEG. The excellent capability for thermal energy storage is attributed to the directional thermal conduction skeletons and perfect open channels and the unique anisotropic three-dimensional structure of the SSPCMs. Hence, the unique structure with PEG is testified as the functional unit for heat storage. Comprehensively considering the excellent properties of sugarcane-based materials—providing cheap raw materials *via* green preparation—it is conceived that sugarcane-based materials could be applied in many energy-related devices with reasonable function unit design.

## Introduction

As we all know, energy plays a very important role in our daily life and energy demand has increased rapidly for both personal and industrial consumption in recent years. (Yu et al., 2020; Wu et al., 2021). The increasing energy shortage and environmental pollution bring an urgency to the improvement of energy storage efficiency and the reduction of greenhouse gas emissions (Huang et al., 2021a; Miyamoto et al., 2020). To solve the above problem, thermal energy storage technology is widely utilized to alleviate the mismatch between energy supply and demand to a certain extent, therefore improving the utilization rate of renewable energy (Lu et al., 2019a; Yang et al., 2019; Li et al., 2022). Solar energy may be one of the most abundant renewable energies to substitute for fossil fuels, which has attracted worldwide attention to heat energy sources for phase change materials (PCMs) (Xi et al., 2014; Zhou et al., 2018; Liu et al., 2020a; 2020b; Wu et al., 2020).

Phase change energy storage technology can be used to solve the problem of energy storage and conservation (Lu et al., 2019b; We et al., 2021). The core of phase change energy storage technology is PCM, whose shape can change and recover within a specific temperature range. For example, polyethylene glycol (PEG) is a widely used and promising solid-liquid PCM (Xie et al., 2020; Hu et al., 2021). But there are two key points that need to be solved urgently. Firstly, liquid leakage in the process of solid-liquid transformation causes pollution to the surrounding environment, so it is limited in practical applications. Secondly, due to the low thermal conductivity of PEG, local high temperatures would occur. In order to solve these problems, the development of composite PCMs with a stable shape and high thermal conductivity has attracted much attention (Yang H. et al., 2018; Zhao et al., 2018; Tony 2020).

Because biomass materials are completely green, cheap and ubiquitous, they have attracted many studies (Huang et al., 2021b). In particular, sugarcane, as a sustainable renewable material, has a unique anisotropic three-dimensional structure (such as a directional thermal conduction skeleton) and perfect open channels (Yang H. et al., 2018; Tangsiriratana et al., 2019; Yang et al., 2020). With low cost and abundant resources, sugarcane is a good choice for a supporting material in composite PCMs. In addition, through certain technological conditions, biomass materials can also be converted into carbon materials that can also be used as supporting materials (Zhao et al., 2018).

In this paper, we creatively propose a method of preparing composite PCMs based on sugarcane with a directional skeleton as support material and PEG as a phase change medium. A series of sugarcane-based composite PCMs were prepared by carbonization and vacuum impregnation for the first time. The effects of carbonization temperature and heating rate on the properties of supporting materials are discussed, which provides a new theoretical basis for the application of sugarcane-based composite PCMs. In addition, this study shows that the sugarcane-based biomass material can be well prepared for the composite PCMs without leakage. Moreover, it can improve its thermal conductivity without adding any additives.

## Experimental Section

The “Experimental section” has four parts: “Materials”, “Preparation of sugarcane-based biomass material”, “Preparation of sugarcane-based biomass/PEG PCMs”, and “Characterization and measurements”. These four parts contain one figure (Supplementary Figure S1) and two tables (Supplementary Tables S1, S2). Here, different sugarcane-based supporting materials can be obtained according to different carbonization processes, which were labeled as NC and 310. And after absorbing PEG, sugarcane-based composite phase change energy storage materials NC/PEG and 310/PEG were obtained. Detailed information from the “Experimental section” is provided in “Supplementary Material”.

## Results and Discussions

### Leakage Test

**FIGURE 1:**
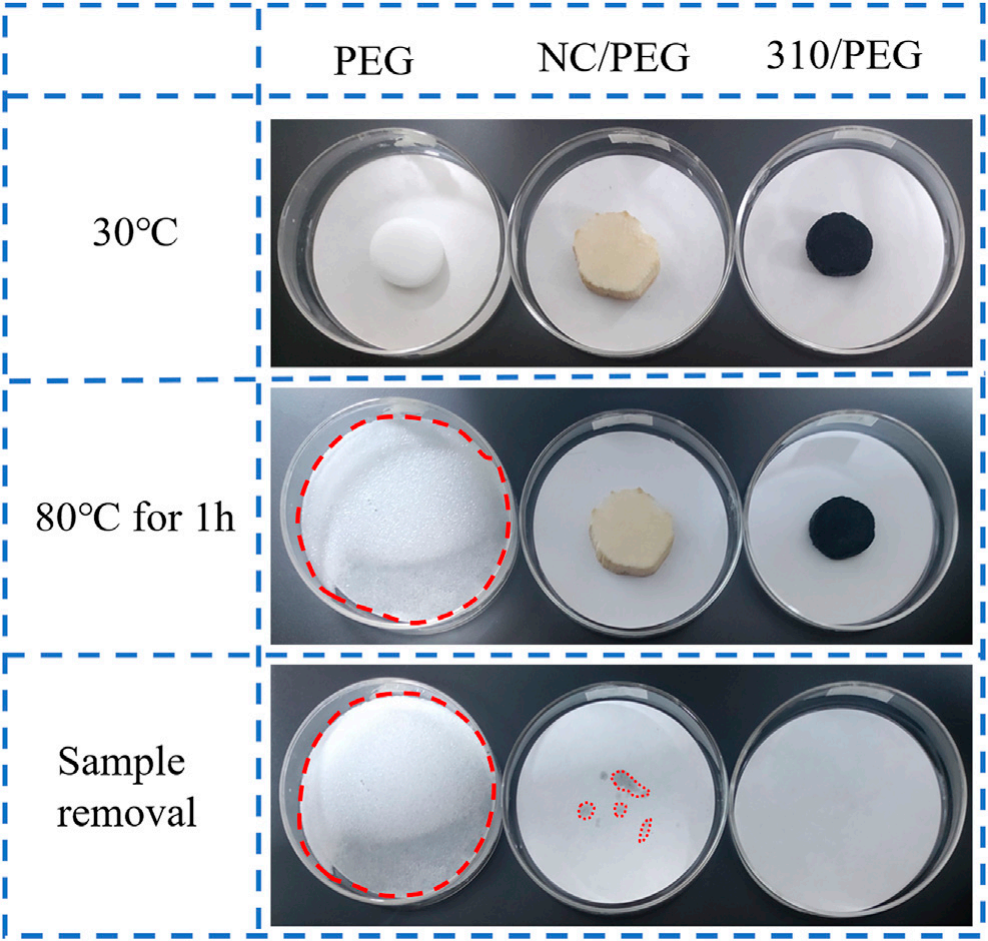
The photographs of PEG, NC/PEG, and 310/PEG composites were heated at 80°C for 1 h.

Figure 1 shows the photographs of the PEG, the NC/PEG, and the 310/PEG composites heated at 80°C for 1 h, which were made into cylindrical samples, and the leakage tests were carried out on filter paper. The samples were heated to 80°C for 1 h to check for leakage of PEG in the composite. Results from digital cameras show that pristine PEG completely melted and flowed at 80°C for 1 h. While NC/PEG and 310/PEG samples did not leak during the whole heating process and had good shape stability. This could be proved by the filter papers after sample removal.

### Microstructure Analysis

**FIGURE 2:**
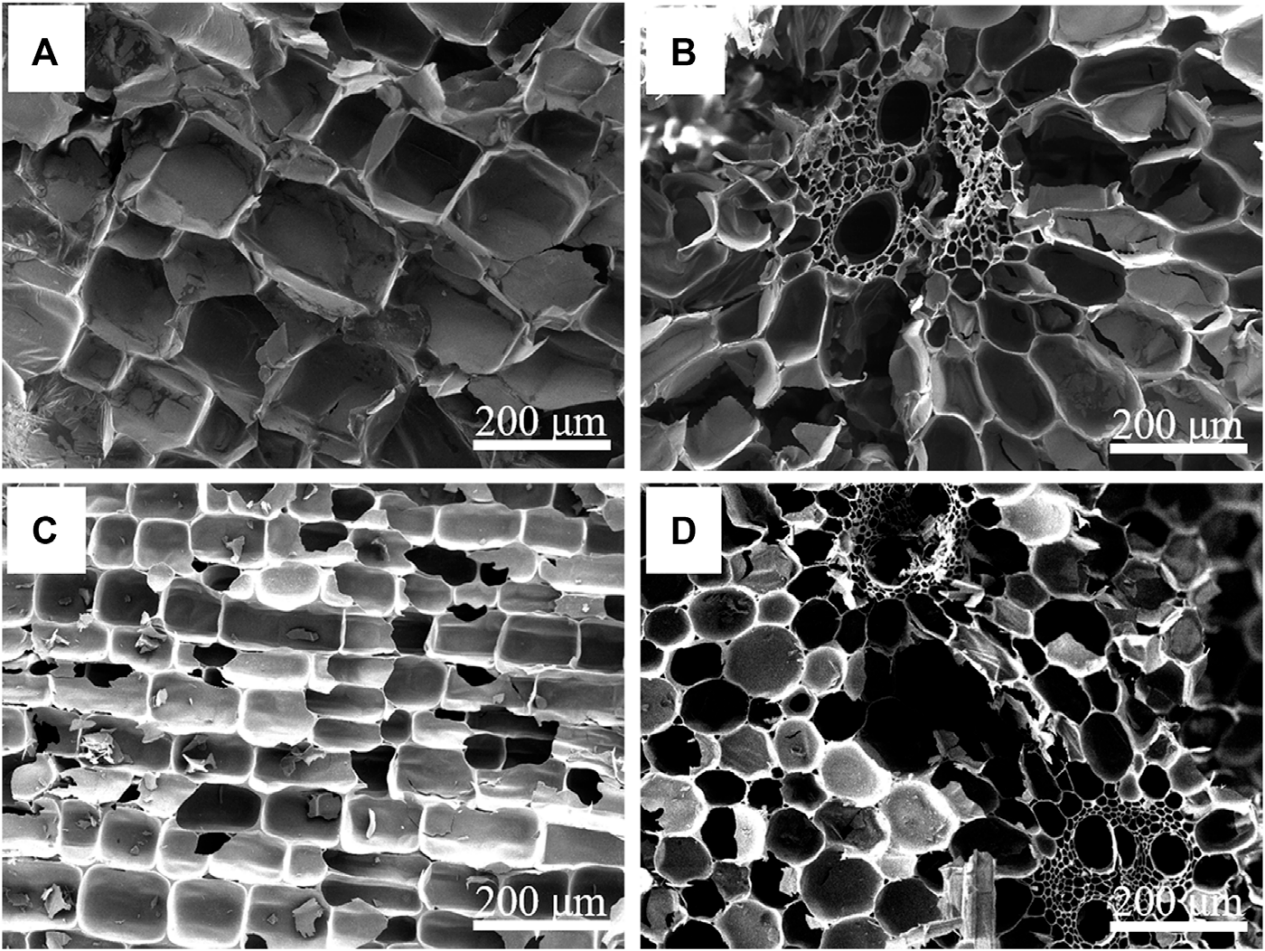
SEM images of longitudinal-sectional and cross-sectional NC **(A,B)** and 310 **(C,D)**.

The previous leakage test results show that NC and 310 as supporting materials of composite PCMs have good shape stability. Therefore, the microstructure of these two kinds of supporting materials and their composite phase change systems were studied. The longitudinal-sectional and cross-sectional morphologies of NC and 310 are shown in Figure 2. As shown in Figures 2A,B, NC has a unique anisotropic three-dimensional porous structure. Figures 2C,D show the morphology of 310, and compared with NC, the morphology of 310 remained good and the pore size was reduced after carbonization, which was consistent with the changes shown in the photos during the leakage test. The perfect open channels and small pits on the inner surface of their cavity help the phase change medium to transport in this continuous porous structure, which is conducive to the preparation of shape stable composite PCMs and their performance as a unit for heat storage.

**FIGURE 3:**
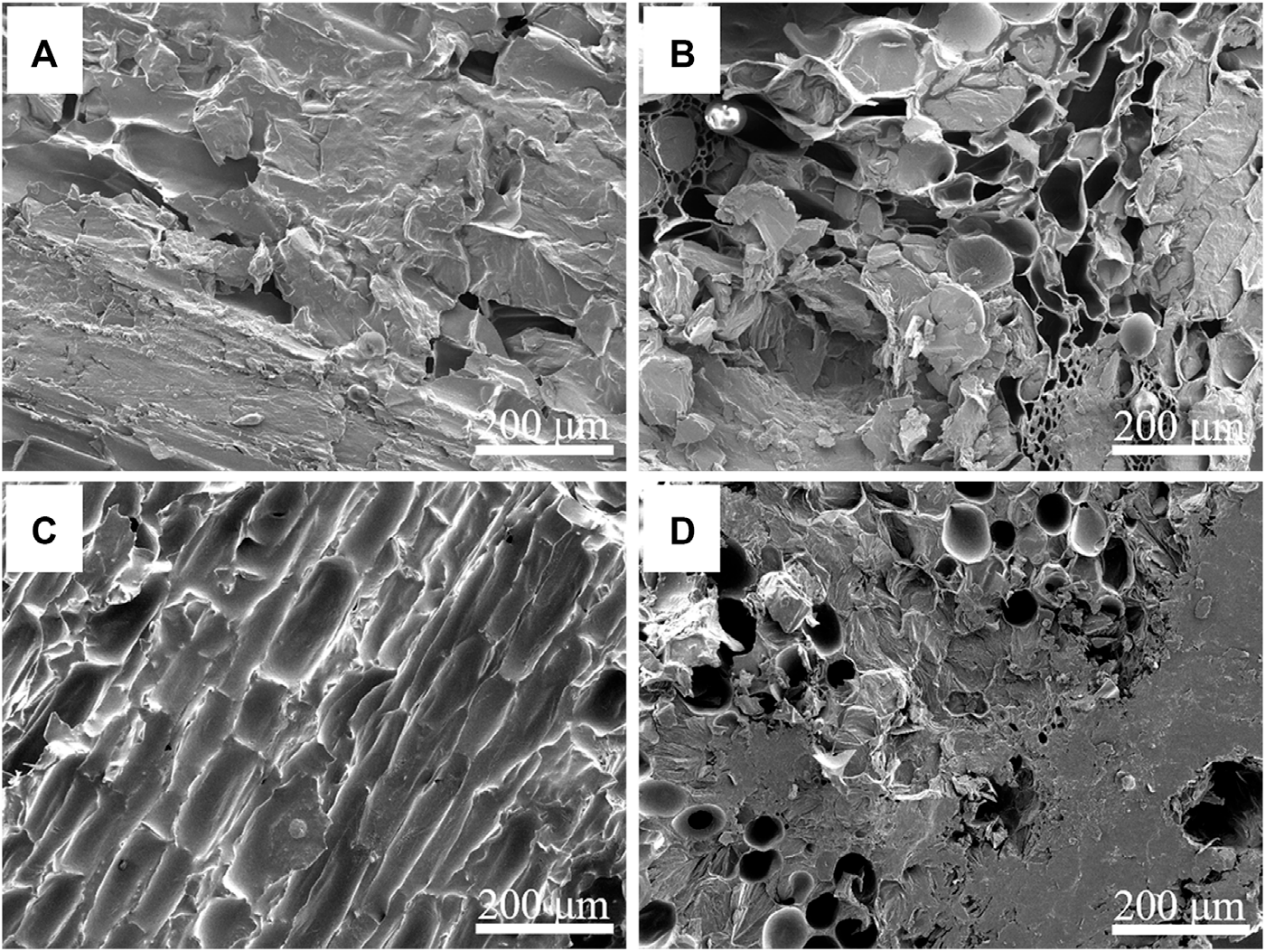
SEM images of longitudinal-sectional and cross-sectional NC/PEG **(A,B)** and 310/PEG **(C,D)**.

The morphologies of the NC/PEG and the 310/PEG longitudinal-sectional and cross-sectional are shown in Figure 3. After vacuum impregnation adsorption, the pores and channels of the NC or the 310 were filled with PEG. Because many closed-pore structures become open-pore structures after carbonization, and some pores are even combined into macropores, the PEG adsorption capacity of the 310 is higher than that of the NC. It can be seen from Figure 3 that the pores of the NC/PEG are not as dense as that of the 310/PEG, which is consistent with Supplementary Table S2. In general, the three-dimensional porous structure prevents leakage of the molten PEG due to capillary effect and surface tension, so that the PEG is successfully encapsulated in the supporting materials. Furthermore, the effect of the 310 is higher than that of the NC, which hints at the importance of the unique structure for heat storage.

### Characterizations of Sugarcane-Based Phase Change Materials

**FIGURE 4:**
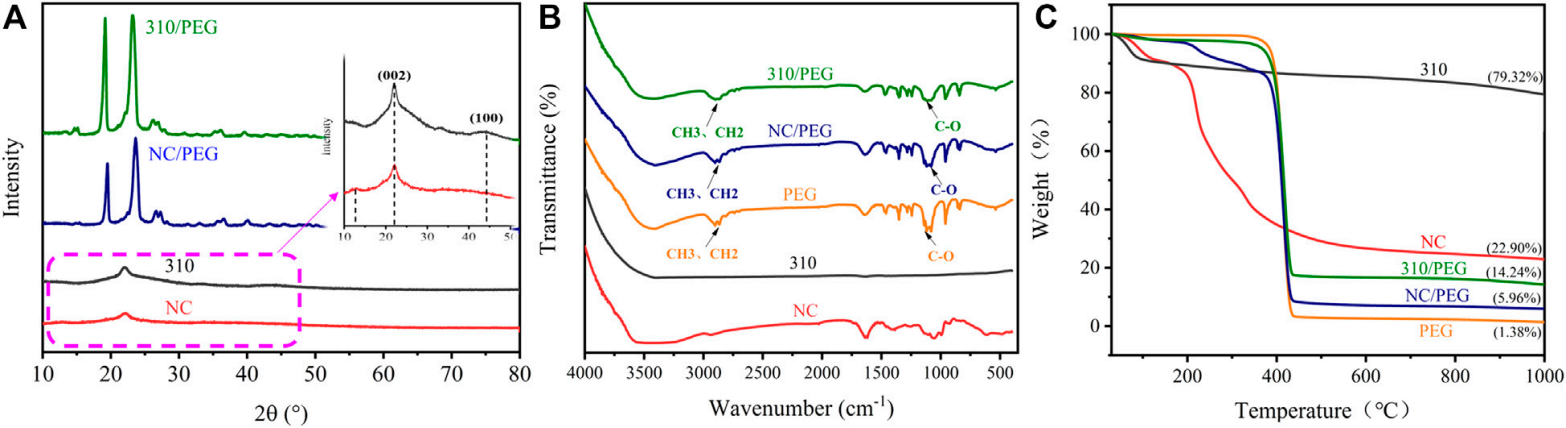
Characterization of samples performed by **(A)** XRD, **(B)** FT-IR, and **(C)** TGA.

In order to verify the chemical structure and adsorption effect of components, NC, 310, PEG, and NC/PEG, 310/PEG were characterized by XRD and FT-IR. The XRD spectra are shown in Figure 4A, there is a certain intensity of diffraction peaks at 2θ = 13° and 2θ = 22° of the NC which are mainly characteristic peaks of cellulose in sugarcane. Compared with the XRD pattern of NC, the XRD pattern of 310 displays a new diffraction peak belonging to 310 that appeared at 2θ = 44°. The diffraction peak at 2θ = 13° that appeared in NC originally has disappeared and the diffraction peak of at 2θ = 22° has on the contrary been strengthened. This result indicates that NC is carbonized to amorphous carbon, the (002) and (100) diffraction peaks of which appear at 2θ = 22° and 44° respectively. The process of carbonization will destroy the crystalline structure of cellulose. The obtained PCMs of NC/PEG and 310/PEG have obvious crystal diffraction peaks of PEG shown in the XRD pattern. The diffraction peaks at 2θ = 19° and 23° correspond to the characteristic diffraction peaks of the (120) and (132) crystal planes of PEG crystals respectively, which related to the strong crystallization ability of PEG and could verify the good adsorption ability of NC and 310, and the crystallization behavior of PEG in PCMs and crystal structure are not affected after compounding.

As Figure 4B shows that, there are no characteristic infrared absorption bands of 310, indicating that NC organic groups have been carbonized and removed owing to 310 being the sugarcane carbonized at 1,000°C. The absorption band at 3,410 cm^−1^ is the tensile vibration of -OH, and 1,100 cm^−1^ belongs to the C-O group absorption peak, which proves the hydroxyl structure of polyethylene glycol. It can be seen from the figure that the absorption peak of PEG and NC/PEG at 2,902 cm^−1^ is the asymmetric stretching vibration of—CH_3_, and the bending vibration with the absorption peak of -CH_3_ at 1,460 cm^−1^ (Pan et al., 2021), which are not found in the FT-IR curves of NC and 310. Compared with pristine PEG, there are no new peaks in the range of 400–4,000 cm^−1^ of NC/PEG and 310/PEG. The above results indicate that the samples NC and 310 are well combined with PEG, and there is only physical adsorption and no chemical at the process of absorption.

In order to characterize the composition of the samples and the thermal stability of PCMs, TGA characterization was performed on it. Figure 4C illuminates the TGA curves of NC, 310, PEG, NC/PEG, and 310/PEG. The thermal decomposition of NC is divided into three stages. The first quality drop at 50–100°C, which is related to the adsorption of air moisture. The second quality drop occurred at 100–220°C. This is because the sucrose inside the NC began to be dehydrated and hydrolyzed at 100°C, and coking and decomposition occurred while the temperature further increased. The third stage of decomposition is the pyrolysis of polysaccharide polymers such as lignin, cellulose, and hemicellulose. As for 310, in addition to the loss of mass which is owed to adsorbing moisture in the air, 310 only slowly loses 12% of its mass. The decomposition curves of NC/PEG and 310/PEG are similar to the decomposition curves of the pristine PEG. Due to the influence of sucrose and plant fiber components in NC, NC/PEG has two mass loss platforms before PEG decomposition, and 5.96% of the mass was retained finally. The decomposition of 310/PEG is similar to PEG, in which degradation temperature is approaching 300°C. However, due to the existence of carbon, 14.24% of the mass was still retained after high-temperature decomposition. The above results indicate that the working temperatures of NC/PEG and 310/PEG are far below the degradation temperature, and show great thermal stability.

### Thermal Properties of Sugarcane-Based Phase Change Materials

**FIGURE 5:**
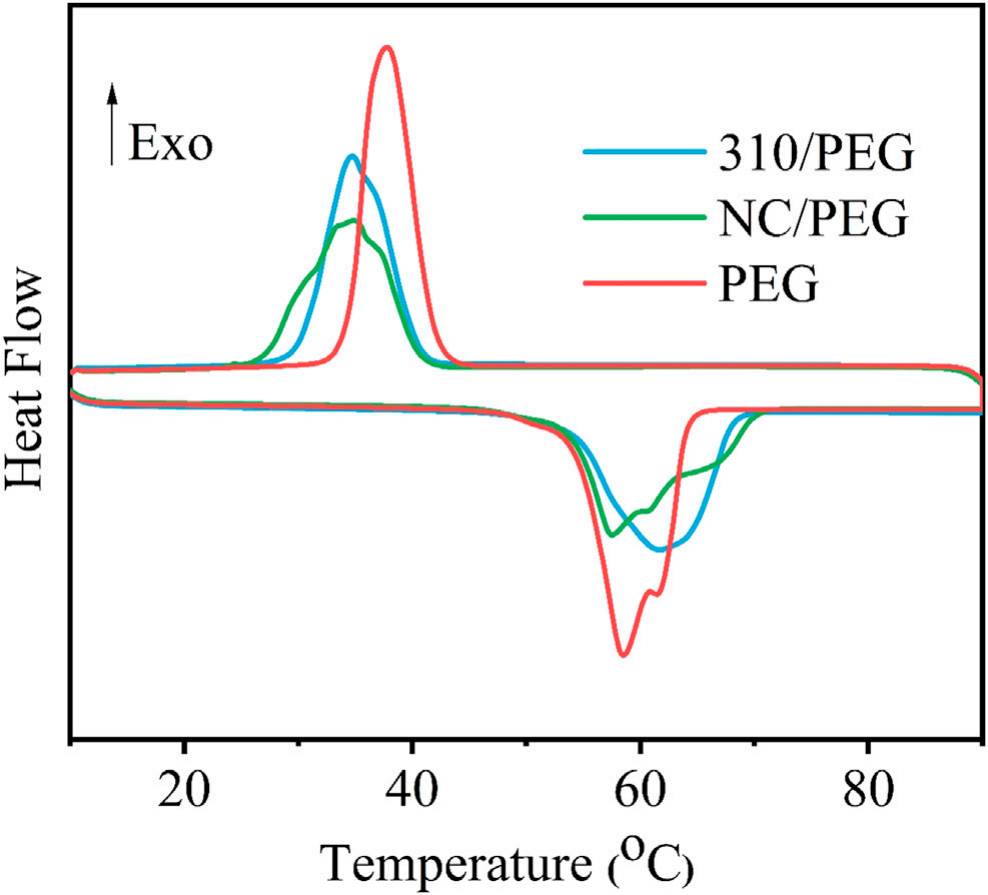
DSC curves of the pristine PEG, the NC/PEG, and the 310/PEG.

Figure 5 shows the DSC curves of the pristine PEG, the NC/PEG, and the 310/PEG, and the corresponding phase change parameters are summarized in Table 1. The thermal behaviors of the pristine PEG, the NC/PEG, and the 310/PEG were investigated in the range of 10–90°C. As shown in Table 1, the Δ*H*_*m*_ and Δ*H*_*c*_ of the NC/PEG and the 310/PEG are lower than those of the pristine PEG. In detail, the total latent heats of melting are 167.5, 130.87, and 140.04 J/g for the PEG, the NC/PEG and the 310/PEG, respectively. The difference between *T*_*m*_ and *T*_*c*_ was within 5°C, which could be attributed to the nucleation rate and spherulite growth rate of PEG segments under the influence of the supporting material.

**TABLE 1:** Phase change properties of PEG, NC/PEG and 310/PEG.

Sample	Δ*H*_*m*_ (J/g)	*T*_*m*_ (°C)	Δ*H*_*c*_ (J/g)	*T*_*c*_ (°C)
PEG	167.5	57.43	163.62	39.07
NC/PEG	130.87	56.97	129.45	35.97
310/PEG	140.04	61.06	137.54	35.59

The relative enthalpy efficiency (*λ*) can be calculated by Eq. 1, which aims to estimate the thermal energy storage capacity of SSPCMs.

$$\text{λ}=\frac{\Delta H_{m-PCM}}{\Delta H_{m-PEG}\times \omega }\times 100\text{\%}$$ (1)

where Δ*H*_*m-PCM*_ and Δ*H*_*m-PEG*_ represent melting heat of SSPCMs and PEG, respectively. And *ω* is the mass fraction of PEG in SSPCMs.

As shown in Table 2, the *λ* values of the NC/PEG and the 310/PEG are 94.84 and 94.39% respectively, which means that the effect on heat storage capacities are small. Compared with previous work (Chen et al., 2011; Liu et al., 2016; Yang Y. et al., 2018; Lu et al., 2019a), the *λ* value of our work is higher. These results show that the addition of sugarcane-based supporting materials would provide a promising method for preparing new bio-based PCMs with high latent enthalpy.

**TABLE 2:** Comparison between the thermal energy storage characteristics of different PEG-based SSPCMs reported in the literature.

SSPCMs	Theoretical enthalpy (J/g)	Latent heat (J/g)	*λ* (%)	References
PEG/PGMA	166.7	73.2	43.9	Chen et al. (2011)
PEG/HDIT	157.0	136.8	87.1	Lu et al. (2019a)
PEG/HDI/CO	167.6	117.7	70.2	Liu et al. (2016)
PEG/MDI/Xylitol	178.1	76.4	42.9	Yang Y. et al. (2018)
NC/PEG	137.99	130.87	94.84	Present study
310/PEG	148.37	140.04	94.39	Present study

Thermal conductivity of sugarcane-based phase change materials.

**FIGURE 6:**
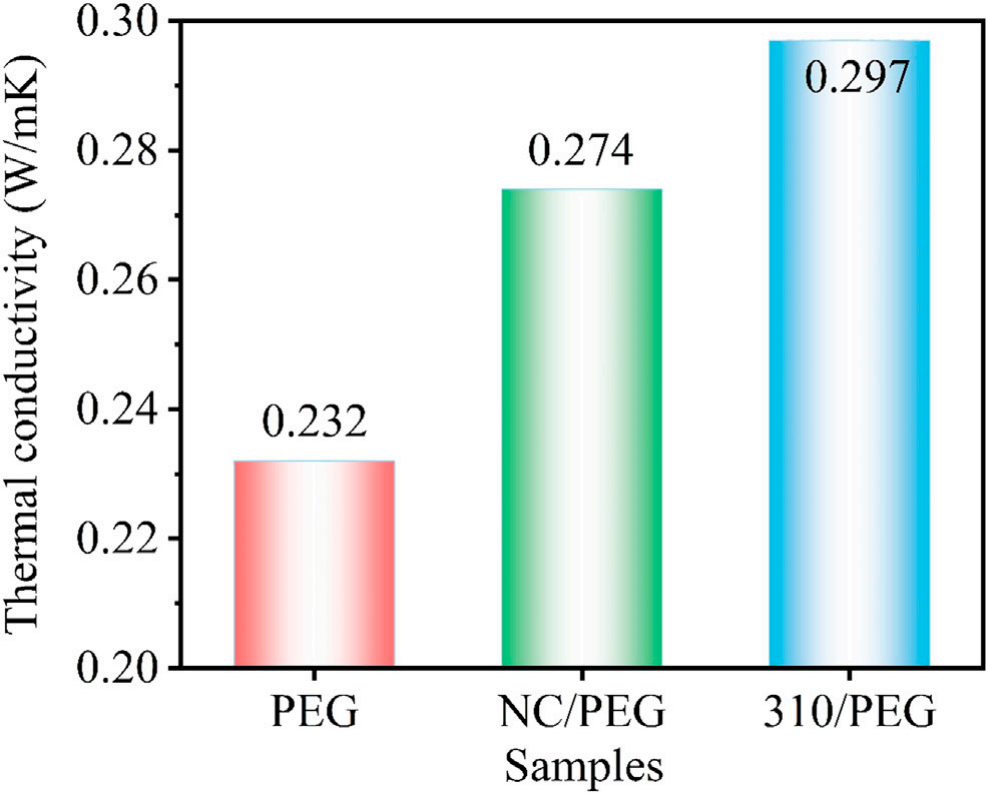
Thermal conductivity of the PEG, the NC/PEG, and the 310/PEG.

Figure 6 shows the thermal conductivity of the PEG, the NC/PEG, and the 310/PEG. It can be seen that the thermal conductivity of pristine PEG is 0.232 W/(mK). NC is a kind of natural biomass material, and the thermal conductivity of its composite NC/PEG is slightly higher than that of pristine PEG, reaching 0.274 W/(mK). The supporting material 310 will have a higher thermal conductivity than NC because of the higher graphitization after carbonization bringing better thermal conductivity (Yang H. et al., 2018). Therefore, the thermal conductivity of the 310/PEG reaches 0.297 W/(mK), which is 28% higher than that of pristine PEG. This is since based on carbonization, sugarcane-based materials also have a high degree of orientation, which is conducive to the improvement of the thermal conductivity of composite materials. Consequently, the heat storage improvement is attributable to the unique structure of the 310/PEG and the pores filled with PEG are the functional unit for heat storage.

## Conclusion

In this study, a series of green and recyclable sugarcane-based PEG composites were prepared by simple carbonization and melt impregnation methods. The excellent thermal energy storage capability was viewed from the unique anisotropic three-dimensional structure of the SSPCMs, including directional thermal conduction skeletons and perfect open channels. The unique structure with PEG was testified as a functional unit for heat storage. The results show that the prepared PCMs have good structure stability, and 310/PEG has stronger adsorption capacity and higher latent heat. Moreover, the highest thermal conductivity of SSPCMs is 0.297 W/(mK), which is 28.02% higher than the pristine PEG. With a combination of low-cost biomass as raw materials, a green production process, and excellent properties, sugarcane-based materials are believed to have promising potential applications in many energy-related devices.

## Data Availability

The original contributions presented in the study are included in the article/Supplementary Material, further inquiries can be directed to the corresponding authors.
