# Supplementary material for: Functional Unit Construction for Heat Storage by Using Biomass-Based Composite
Source: Front Chem. 2022 Feb 7;10:835455. doi: 10.3389/fchem.2022.835455 (PMC8859462; doi:10.3389/fchem.2022.835455)
Supplement: Supplementary file 1 [file DataSheet1.pdf]

Supporting information

Functional unit constructing for heat storage by using biomass-based composite

Jingtao Su[1], Mengman Weng[1], Xiang Lu[2,3], Weihao Xu[1], Sha Lyu[4,*], Yidong Liu[1], Yonggang Min[1,*]

[1] Department of Polymeric Materials and Engineering, School of Materials and Energy, Guangdong University of Technology, Guangzhou, 510006, China

[2] Key Laboratory of Polymer Processing Engineering (South China University of Technology), Ministry of Education, Guangzhou, 510640, China

[3] Key Laboratory of Material Chemistry for Energy Conversion and Storage of Ministry of Education, School of Chemistry and Chemical Engineering, Huazhong University of Science & Technology, Wuhan, 430074, China

[4] Department of Materials Science and Engineering, Southern University of Science and Technology, Shenzhen, 518055, China

*Email: lus@sustech.edu.cn (S. L.), ygmin@gdut.edu.cn (Y.M.).

EXPERIMENTAL SECTION

Materials

The PEG (M_w = 4000) with purity in mass fractions larger than 0.98 used in our study was received from Macklin. Fresh sugarcane was purchased from the market.

TABLE S1 | Different numbers of sugarcane-based biomass materials under different temperatures and rates.

Temperature (°C)	Rate (°C/min)	Sample
Non carbonized	Non carbonized	NC
600	3	36
800	3	38
1000	3	310
1000	5	510
1000	10	1010

Preparation of sugarcane-based biomass material

The sugarcane-based biomass materials were synthesized through a sample carbonation process, following the schematic diagram in **Figure S1(a)**. Fresh sugarcane was cut into pieces. To obtain dehydrated specimens, fresh pieces were frozen for 6 h and then dried for 72 h in the vacuum freeze dryer. Samples were then heated in a tube furnace at different temperatures (600, 800, 1000 °C), under N_2 atmosphere at different rates (3, 5 and 10 °C /min) for 2h (**Table S1**).

TABLE S2 | The proportion of PEG in samples.

Sample	The proportion of PEG (%)
NC/PEG	82.38
36/PEG	83.79
38/PEG	84.67
310/PEG	88.58
510/PEG	82.64
1010/PEG	84.52

Preparation of sugarcane-based biomass/PEG PCMs

The sugarcane-based biomass/PEG PCMs were synthesized through a vacuum impregnation process, following the schematic diagram in **Figure S1(b)**. Weigh the mass of the corresponding sugarcane-based sample, then place enough PEG into several glass beakers respectively, keep it in an 80 °C oven for 1 h, and immerse the sugarcane-based sample completely after the PEG is completely melted. After adequate coverage and immersion, the different glass beakers of sugarcane-based/PEG mixtures were

heated in a vacuum oven at 80 °C for 24 h under the vacuum of −0.1 kPa. Then several leakage experiments were carried out until the weight did not change. Finally, sugarcane-based composite phase change energy storage materials NC/PEG, 36/PEG, 38/PEG, 310/PEG, 510/PEG and 1010/PEG were obtained. The proportion of PEG in the samples is shown in **Table S2**.

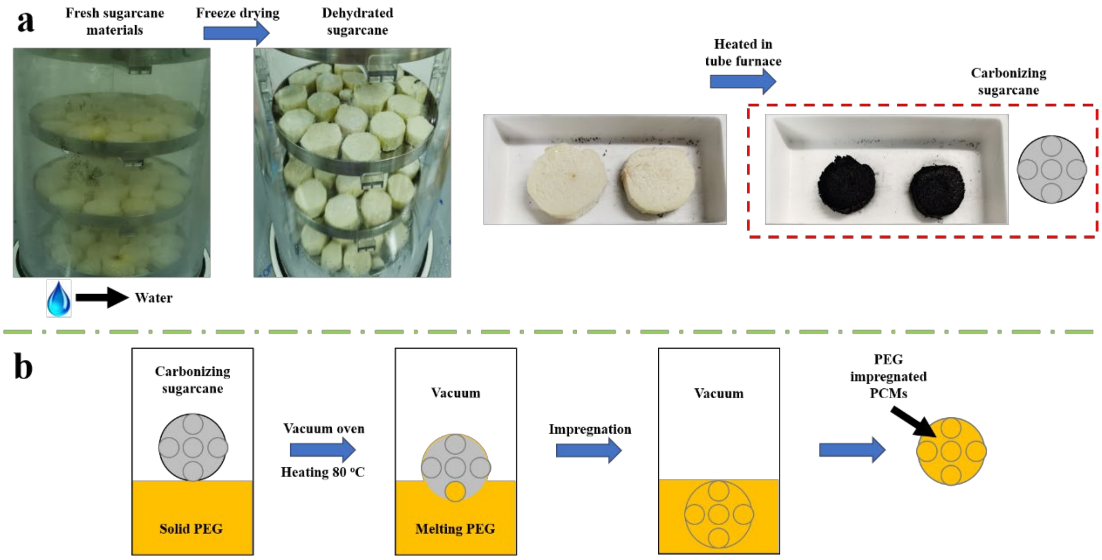

FIGURE S1 | Schematic diagram of sugarcane-based biomass materials synthesis (a) and PCMs synthesis.

Characterization and measurements

A Tescan VEGA 3 LMH scanning electron microscope (SEM) equipped at an accelerating voltage of 10 kV was used to characterize the samples. Before the SEM observations, all samples were coated with a thin film of platinum (Pt) to enhance the imaging contrast. The characterization of chemical structure and composition of all samples were performed by X-ray diffractometer (XRD) and Fourier transform infrared spectrometer (FT-IR). Among them, XRD, (Panalyticial Aeris) with Cu K_α radiation (40kV, 15 mA) adopt the way of slow scan at a scanning rate of 2°/min from 10° to 80°. FT-IR, (Fourier Nicolet 6700, USA) adopt the ATR strategy at the wavenumber range of 400 cm^{-1} to 4000 cm^{-1} and the samples were prepared by the method of KBr tablet pressing. In the meantime, the thermal stability of samples was evaluated on about 10 mg samples by using a star system thermogravimetric analysis (TGA, Mettler

Toledo Ltd., USA) between 30 °C and 1000 °C in a nitrogen atmosphere (50 ml/min) with a 10 °C min^{-1} heating ramp. All thermal parameters were determined as the average of three experiments. The phase change properties, including the phase change temperatures and phase change enthalpies, of all samples, were determined by a differential scanning calorimetry (DSC, METTLER DSC 1) from 0 °C to 100 °C at a heating/cooling rate of 10 °C/min under a nitrogen atmosphere. The measurement of thermal conductivity of the samples was conducted using a hot disk thermal constant analyzer (TPS 2500S, Sweden) with the C7577 probe at room temperature. For the repeatability of the results, at least five measurements were performed for each sample.
